# Performance of predictive AI-based clinical decision support systems across clinical domains: A systematic review and meta-analysis

**DOI:** 10.1371/journal.pdig.0001310

**Published:** 2026-03-24

**Authors:** William J. Waldock, Ahmad Guni, Ara Darzi, Hutan Ashrafian

**Affiliations:** Institute of Global Health Innovation, Imperial College London, London, United Kingdom; National Taichung University of Science and Technology, TAIWAN

## Abstract

Despite advances in deep learning and transformer architectures, prior reviews have focused narrowly on traditional clinical decision support systems (CDSS) or single medical domains, leaving significant gaps in understanding contemporary AI-driven predictive tools. This systematic review and meta-analysis evaluated the predictive performance of artificial intelligence-based CDSS (AI-CDSS) across multiple medical specialties. Following PRISMA guidelines, PubMed and Cochrane Library were searched through December 2024 for studies evaluating predictive AI-CDSS using real-world clinical data. Two reviewers independently screened 3,296 records (κ = 0.833), with study quality assessed via QUADAS-2 and performance measures pooled using random-effects meta-analysis. Fifty studies spanning 17 medical specialties were included. Meta-analysis demonstrated moderate discriminatory ability (pooled AUC: 0.652, 95% CI: 0.562–0.743), high specificity (0.819, 95% CI: 0.793–0.844), moderate accuracy (0.765, 95% CI: 0.734–0.796), and variable sensitivity (0.660, 95% CI: 0.535–0.785), with substantial heterogeneity across all measures (I² ≥ 98.9%). Only 24% of studies involved prospective deployment, and 64% reported exclusively technical metrics without clinical workflow data. Predictive AI-CDSS demonstrate moderate-to-good diagnostic performance with strong specificity; however, the predominance of retrospective study designs and limited implementation reporting reveal critical gaps between technical validation and real-world clinical utility. To address these shortcomings, we propose the ROADMAP framework, structured around seven domains: Representative development, Outcomes-focused evaluation, Assessment for deployment, Data harmonization, Monitoring for bias, Allocation via economic evaluations, and Priorities for standardized reporting and prospective validation. This framework provides a practical roadmap for bridging the gap between algorithmic performance and meaningful clinical integration.

## Author summary

In our study, we set out to understand how well modern Artificial Intelligence (AI) assists doctors in making clinical decisions across a wide range of medical specialties. While AI technology has advanced rapidly, we realized that previous research was often too narrow or outdated to show the full picture of these modern predictive tools.

After reviewing 50 studies covering 17 different medical fields, we found that current AI tools demonstrate moderate to good accuracy. They are particularly effective at correctly identifying when a patient *does not* have a condition (high specificity). However, they are less consistent at catching every positive case, and their performance varies significantly depending on the setting.

Crucially, we identified a major gap between technical success and real-world usefulness. Most studies tested AI on historical data rather than in live hospital environments, often ignoring how these tools fit into a doctor’s actual workflow.

To address this, we developed the ROADMAP framework when applying our findings to the case study to antimicrobial resistance. This seven-step guide outlines how researchers can move beyond simple math scores to create AI tools that are representative, fair, economically viable, and proven to work in actual patient care scenarios.

## Background

Artificial Intelligence (AI), encompassing a wide spectrum of computational approaches, has transformed clinical decision-making in healthcare by enhancing predictive capabilities and enabling precise, data-driven interventions [1]. The landscape of clinical decision support has evolved rapidly with recent advances in AI methodologies, particularly deep learning architectures, convolutional neural networks, and transformer-based models. These modern AI techniques have demonstrated unprecedented capacity to identify complex patterns in clinical data, generating predictions that inform diagnostic, prognostic, and therapeutic decisions.

Despite the proliferation of AI-CDSS development and the growing body of literature reporting technically promising but with limited evidence of real‑world impact, several critical knowledge gaps persist. First, previous systematic reviews have primarily focused on traditional CDSS or have been limited to single clinical domains such as cardiology, oncology, or radiology [2–4], lacking comprehensive synthesis across the diverse landscape of AI-driven predictive tools. Second, the rapid evolution of AI methodologies, particularly the emergence of deep learning and transformer-based architectures in recent years [5], necessitates contemporary evaluation that reflects current technological capabilities. Third, significant heterogeneity exists in how AI-CDSS performance is evaluated and reported, with inconsistent use of metrics and validation approaches across studies [6]. This variability complicates efforts to assess the true clinical utility of these tools and compare performance across different systems and clinical contexts.

Furthermore, concerns regarding explainability, clinical integration, and liability have been identified as barriers to frontline clinical adoption [7]. While AI models may achieve high technical performance in controlled settings, questions remain about their real-world effectiveness, including how well they integrate into clinical workflows, whether clinicians trust and adopt their recommendations, and whether they ultimately improve patient outcomes. The gap between technical validation and clinical implementation represents a critical consideration for the field.

The need for rigorous, comprehensive evaluation of AI-CDSS spans multiple clinical domains. While specific applications, such as antimicrobial stewardship, where prescription surveillance has been implemented in Australia [8], Japan [9], and Africa [10], demonstrate the potential impact of decision support tools, the broader landscape of predictive AI-CDSS warrants systematic examination. A previous systematic review examining traditional Clinical Decision Support Systems and their role in antibiotic stewardship [11] found that CDSS interventions significantly improved outcomes relevant to antibiotic prescribing, with both active and passive systems contributing to more appropriate antibiotic use and improved patient outcomes. However, this work focused specifically on antibiotic stewardship and did not comprehensively evaluate modern AI-driven predictive models across diverse clinical domains. Unlike domain-specific reviews of AI-CDSS in nursing, psychiatry, obstetrics, and oncology, our review delivers the first multi-domain synthesis of predictive AI-CDSS performance across more than 15 specialties using standardised metrics, explicitly excluding rule-based systems and non-predictive tools examined in prior work. We uniquely quantify the critical implementation gap, demonstrating that while AI-CDSS show strong technical performance, evidence for clinical integration remains severely limited.

## Objective

This study aims to systematically evaluate the predictive performance of AI-based clinical decision support systems (AI-CDSS) across a broad range of medical domains. By synthesizing evidence on diagnostic accuracy, prognostic capability, and, where reported, clinical implementation metrics, we seek to:

1. Quantify the pooled diagnostic performance of predictive AI-CDSS using standardized metrics (sensitivity, specificity, accuracy, and AUROC)
2. Assess the methodological quality and risk of bias in AI-CDSS evaluation studies
3. Identify sources of heterogeneity in reported performance across clinical domains, patient populations, and AI methodologies
4. Inform the potential and limitations of predictive AI-CDSS for integration into routine clinical practice across diverse healthcare settings

This multi-domain synthesis provides a foundation for understanding the current state of predictive AI-CDSS development and validation, highlighting both the promise of these technologies and the critical needs for standardized evaluation methodologies and real-world clinical assessment.

## Results

### Study selection and screening process

**Fig 1:**
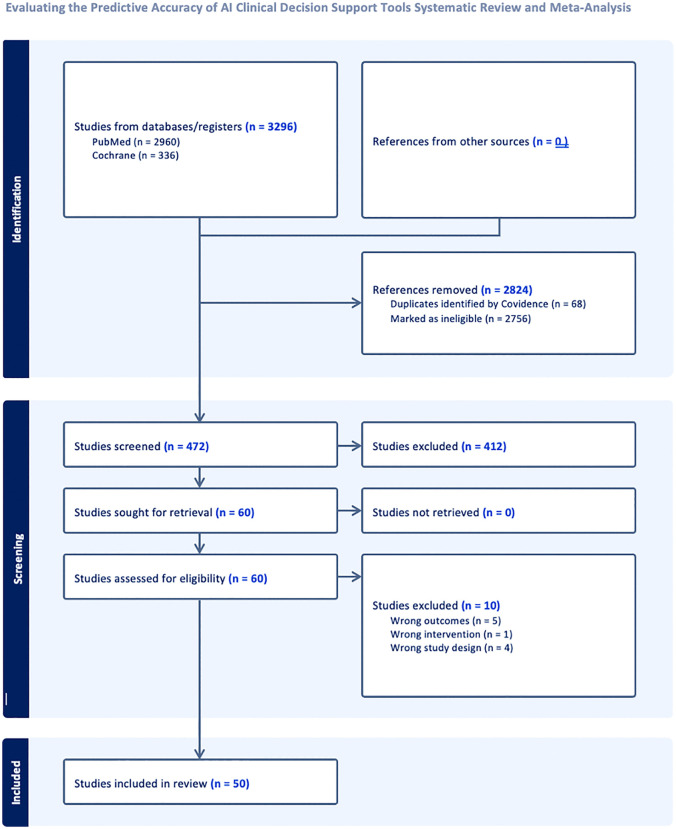
PRISMA diagram.

This systematic review adheres to the PRISMA (Preferred Reporting Items for Systematic Reviews and Meta-Analyses) [12] guidelines to ensure transparency and methodological rigor. The study selection process is summarized in the PRISMA flow diagram (Fig 1), with Supplementary PRISMA Checklist (S1 File). A total of 3,296 studies were identified through database searches (PubMed: 2,960; Cochrane: 336). After removing 2,824 studies due to duplication or clear ineligibility, 472 abstracts were screened in detail. Of these, 60 studies were retrieved for full-text assessment, and 50 met the inclusion criteria and were included in the final analysis (Tables 1,2).

**Table 1:** Study characteristics.

	Study ID	Specialty	Country	Title	Patients
1	Bang 2022	ONC	South Korea	Deep-Learning-Based Clinical Decision Support System for Gastric Neoplasms in Real-Time Endoscopy: development and Validation Study	2524
2	Bertsimas 2021	PAEDS	Turkey	Selecting Children with Vesicoureteral Reflux Who are Most Likely to Benefit from Antibiotic Prophylaxis: application of Machine Learning to RIVUR	607
3	Bhagawati 2024	CVD	Canada	Deep learning approach for cardiovascular disease risk stratification and survival analysis on a Canadian cohort.	459
4	Bolton 2024	ID	UK	Personalising intravenous to oral antibiotic switch decision making through fair interpretable machine learning	10362
5	Cha 2019	URO	USA	Diagnostic Accuracy of CT for Prediction of Bladder Cancer Treatment Response with and without Computerized Decision Support.	123
6	Connor 2007	GASTRO	Australia	The application of machine learning techniques as an adjunct to clinical decision making in alcohol dependence treatment.	66
7	Corbin 2022	ID	USA	Personalized antibiograms for machine learning driven antibiotic selection	24148
8	Du 2022	ENDO	Ireland	An explainable machine learning-based clinical decision support system for prediction of gestational diabetes mellitus.	77
9	Feretzakis 2021	ID	Greece	Machine Learning for Antibiotic Resistance Prediction: A Prototype Using Off-the-Shelf Techniques and Entry-Level Data to Guide Empiric Antimicrobial Therapy	499
10	Gomez 2024	ENT	USA	Explainable AI decision support improves accuracy during telehealth strep throat screening.	121
11	Gomez-Cabello 2024	PLAST	USA	Large Language Models for Intraoperative Decision Support in Plastic Surgery: A Comparison between ChatGPT-4 and Gemini.	32
12	Han 2020	CVD	South Korea	Machine learning based risk prediction model for asymptomatic individuals who underwent coronary artery calcium score: Comparison with traditional risk prediction approaches.	690
13	Hebert 2020	ID	USA	Prediction of Antibiotic Susceptibility for Urinary Tract Infection in a Hospital Setting	6366
14	Hirosawa 2024	CVD	Japan	Clinical decision support system using a machine learning model to assist simultaneous cardiopulmonary auscultation: open-label randomized controlled trial	384
15	Hoffer 2024	ENT	Israel	Machine Learning for Clinical Decision Support of Acute Streptococcal Pharyngitis: A Pilot Study.	54
16	Hou 2020	ED	China	Predicting 30-days mortality for MIMIC-III patients with sepsis-3: a machine learning approach using XGboost.	4559
17	Jia 2024	RESP	China	Deep learning prediction of survival in patients with heart failure using chest radiographs.	353
18	Kanjilal 2020	ID	USA	A decision algorithm to promote outpatient antimicrobial stewardship for uncomplicated urinary tract infection	3629
19	Keim-Malpass 2024	CVD	USA	Prospective validation of clinical deterioration predictive models prior to intensive care unit transfer among patients admitted to acute care cardiology wards.	10422
20	Lamping 2018	ED	Germany	Development and validation of a diagnostic model for early differentiation of sepsis and non-infectious SIRS in critically ill children - a data-driven approach using machine-learning algorithms.	58
21	Lee 2021	ID	Hong Kong	Deep learning model for prediction of extended-spectrum beta-lactamase (ESBL) production in community-onset Enterobacteriaceae bacteraemia from a high ESBL prevalence multi-centre cohort	5626
22	Letterie 2020	OBGYN	USA	Artificial intelligence in in¬†vitro fertilization: a computer decision support system for day-to-day management of ovarian stimulation during in¬†vitro fertilization.	2603
23	Lewin-Epstein 2021	ID	Israel	Predicting Antibiotic Resistance in Hospitalized Patients by Applying Machine Learning to Electronic Medical Records	4360
24	Li 2023	CVD	China	Development and Validation of Machine Learning-Based Models to Predict In-Hospital Mortality in Life-Threatening Ventricular Arrhythmias: retrospective Cohort Study	3140
25	Liang 2022	ID	China	Early prediction of carbapenem-resistant Gram-negative bacterial carriage in intensive care units using machine learning	2920
26	Liu 2023	GASTRO	China	Construction and validation of machine learning models for sepsis prediction in patients with acute pancreatitis.	1672
27	McGonagle 2023	PAEDS	USA	Evaluation of an Antimicrobial Stewardship Decision Support for Pediatric Infections.	21
28	Nau 2020	CVD	USA	A Machine Learning Based Risk Stratification Tool to Coordinate Referrals Between Inpatient Specialty and Palliative Care for Patients with Heart Failure (RP320)	5676
29	Oonsivilai 2019	ID	Thailand	Using machine learning to guide targeted and locally-tailored empiric antibiotic prescribing in a children’s hospital in Cambodia	243
30	Papachristou 2024	DERM	Sweden	Evaluation of an artificial intelligence-based decision support for the detection of cutaneous melanoma in primary care: a prospective real-life clinical trial.	228
31	Pearce 2019	ED	Australia	POLAR Diversion: Using General Practice Data to Calculate Risk of Emergency Department Presentation at the Time of Consultation.	744477
32	Prelaj 2022	ONC	Italy	Real-world data to build explainable trustworthy artificial intelligence models for prediction of immunotherapy efficacy in NSCLC patients.	480
33	Rawson 2021	ID	UK	A Real-world Evaluation of a Case-based Reasoning Algorithm to Support Antimicrobial Prescribing Decisions in Acute Care	224
34	Rich 2022	ID	USA	Development of a Prediction Model for Antibiotic-Resistant Urinary Tract Infections Using Integrated Electronic Health Records from Multiple Clinics in North-Central Florida	9990
35	Rojas 2024	RENAL	Colombia	Development and validation of interpretable machine learning models to predict glomerular filtration rate in chronic kidney disease Colombian patients.	29000
36	Sadik 2006	ORTHO	Sweden	A new computer-based decision-support system for the interpretation of bone scans.	200
37	ShahryariFard 2024	HAEM	Canada	A deep-learning approach to predict bleeding risk over time in patients on extended anticoagulation therapy.	2542
38	Sick-Samuels 2020	ID	USA	A Decision Tree Using Patient Characteristics to Predict Resistance to Commonly Used Broad-Spectrum Antibiotics in Children With Gram-Negative Bloodstream Infections	689
39	Simmons 2024	ORTHO	USA	Initial clinical experience with a predictive clinical decision support tool for anatomic and reverse total shoulder arthroplasty.	243
40	Solomon 2020	CVD	USA	Forecasting a Crisis: Machine-Learning Models Predict Occurrence of Intraoperative Bradycardia Associated With Hypotension.	3498
41	Sun 2023	ONC	China	Prediction models for chronic postsurgical pain in patients with breast cancer based on machine learning approaches	1152
42	Taneja 2017	ED	USA	Combining Biomarkers with EMR Data to Identify Patients in Different Phases of Sepsis.	444
43	Tzelves 2022	ID	Greece	Using machine learning techniques to predict antimicrobial resistance in stone disease patients	239
44	Vaid 2023	ED	USA	Implications of the Use of Artificial Intelligence Predictive Models in Health Care Settings: A Simulation Study.	130000
45	Wang 2021	CVD	China	Application of machine learning to predict the occurrence of arrhythmia after acute myocardial infarction.	2084
46	Yang 2020	OBGYN	China	Predictive models of hypertensive disorders in pregnancy based on support vector machine algorithm.	690
47	Yelin 2019	ID	Israel	Personal clinical history predicts antibiotic resistance of urinary tract infections	315047
48	Yoon 2020	VASC	South Korea	MED-TMA: A clinical decision support tool for differential diagnosis of TMA with enhanced accuracy using an ensemble method.	319
49	Zeng 2023	ONC	China	Development and validation of survival prediction model for gastric adenocarcinoma patients using deep learning: a SEER-based study	14177
50	Zhang 2024	RENAL	China	Automated machine learning for early prediction of acute kidney injury in acute pancreatitis.	437

**Table 2:** Study results.

Study ID	Patients	AUROC	Accuracy	Sensitivity	Specificity	AUC
Bang 2022	2524	–	0.815	–	–	–
Bertsimas 2021	607	–	–	–	–	0.82
Bhagawati_BiGAN 2024	459	–	0.8662	0.8812	0.9178	0.929
Bhagawati_BiGRU 2024	459	–	0.8442	0.8518	0.8317	0.908
Bhagawati_BiLSTM 2024	459	–	0.8632	0.8612	0.8529	0.916
Bhagawati_BiRNN 2024	459	–	0.8322	0.8434	0.8216	0.896
Bhagawati_GAN 2024	459	–	0.8301	0.8634	0.9113	0.916
Bhagawati_GRU 2024	459	–	0.8118	0.8317	0.8532	0.895
Bhagawati_LSTM 2024	459	–	0.8288	0.8437	0.8767	0.91
Bhagawati_RNN 2024	459	–	0.801	0.8288	0.8496	0.886
Bolton 2024	10362	0.8	–	–	–	–
Cha 2019	123	–	–	–	–	0.77
Connor 2007	66	–	0.77	–	–	–
Corbin 2022	24148	0.74	–	–	–	–
Du_1 2022	77	0.86	–	0.833	0.754	0.551
Du_2 2022	77	0.69	–	0.583	0.6	0.256
Du_3 2022	77	0.687	–	0.5	0.708	0.32
Feretzakis 2021	499	0.822	–	–	–	–
Gomez 2024	121	–	–	0.65	0.77	–
Gomez-Cabello_ChatGPT-4 2024	32	–	0.56	–	–	–
Gomez-Cabello_Gemini 2024	32	–	0.28	–	–	–
Gong 2023	5017	–	0.815	–	–	–
Han_ASCVD 2020	690	–	–	–	–	0.72
Han_FRS 2020	690	–	–	–	–	0.62
Han_LR 2020	690	–	–	–	–	0.74
Han_ML 2020	690	–	–	–	–	0.78
Hebert 2020	6366	0.69	–	–	–	–
Hirosawa 2024	384	–	0.893	–	–	–
Hoffer 2024	54	–	–	–	–	–
Hou_SAPS 2020	4559	–	–	–	–	0.797
Hou_Trad 2020	4559	–	–	–	–	0.819
Hou_XGBoost 2020	4559	–	–	–	–	0.857
Jia_CLINICALCOX 2024	353	–	0.674	0.583	0.724	–
Jia_DLSPCXR 2024	353	–	0.757	0.604	0.845	–
Jia_DLSPinteg 2024	353	–	0.818	0.75	0.776	–
Jia_IMAGINGCOX 2024	353	–	0.561	0.5	0.655	–
Kanjilal 2020	3629	0.64	–	–	–	–
Keim-Malpass_AllCause 2024	10422	–	–	–	–	0.733
Keim-Malpass_CardioResp 2024	10422	–	–	–	–	0.737
Keim-Malpass_CVD 2024	10422	–	–	–	–	0.725
Lamping 2018	58	–	–	–	–	0.78
Lee 2021	5626	0.761	–	–	–	–
Letterie 2020	2603	–	0.92	0.94	–	–
Lewin-Epstein 2021	4360	0.88	–	–	–	–
Li_CatBoost 2023	3140	–	–	–	–	0.905
Li_LightGBM 2023	3140	–	–	–	–	0.901
Li_LODS 2023	3140	–	–	–	–	0.749
Li_OS_DT 2024	273	–	0.61	–	–	0.608
Li_OS_LR 2024	273	–	0.573	–	–	0.628
Li_OS_XGB 2024	273	–	0.634	–	–	0.695
Li_PFS_DT 2024	273	–	0.549	–	–	0.696
Li_PFS_LR 2024	273	–	0.524	–	–	0.511
Li_PFS_XGB 2024	273	–	0.565	–	–	0.677
Li_SAPS-II 2023	3140	–	–	–	–	0.78
Liang 2022	2920	0.91	–	–	–	–
Liu 2023	1672	–	–	–	–	0.985
McGonagle 2023	21	–	–	0.73	0.636	
Nau 2020	5676	–	–	–	–	0.809
Oonsivilai 2019	243	0.85	–	–	–	–
Papachristou 2024	228	0.96	–	–	–	–
Pearce 2019	744477	–	–	0.96	–	–
Prelaj_CB 2022	480	–	–	–	–	0.75
Prelaj_LR 2022	480	–	–	–	–	0.77
Rawson 2021	224	–	0.83	–	–	–
Rich 2022	9990	0.66	–	–	–	–
Rojas 2024	29000	–	–	–	–	–
Sadik 2006	200	–	–	0.9	0.74	–
ShahryariFard_ACCP 2024	2542	0.667	0.696	0.606	0.7	0.081
ShahryariFard_ANN 2024	2542	0.771	0.854	0.424	0.874	0.113
ShahryariFard_ANN2024	2542	0.612	0.743	0.515	0.753	0.072
ShahryariFard_CHAP 2024	2542	0.681	0.743	0.545	0.752	0.188
ShahryariFard_Ensemble 2024	2542	0.824	0.808	0.606	0.817	0.141
ShahryariFard_FUP 2024	2542	0.807	0.76	0.758	0.76	0.129
ShahryariFard_HAS-BLED 2024	2542	0.642	0.908	0.333	0.935	0.135
ShahryariFard_OBRI 2024	2542	0.663	0.944	0.03	0.986	0.08
ShahryariFard_Overall 2024	2542	–	–	0.61	0.82	–
ShahryariFard_RIETE 2024	2542	0.615	0.951	0	0.994	0.063
ShahryariFard_VTE-BLEED 2024	2542	0.651	0.74	0.485	0.752	0.069
Sick-Samuels 2020	689	0.7	–	–	–	–
Simmons_Abduction 2024	243	0.72	–	–	–	–
Simmons_ASES 2024	243	0.87	–	–	–	–
Simmons_Constant 2024	243	0.89	–	–	–	–
Simmons_Elevation 2024	243	0.74	–	–	–	–
Simmons_Global 2024	243	0.83	–	–	–	–
Simmons_IR 2024	243	0.8	–	–	–	–
Simmons_Rotation 2024	243	0.73	–	–	–	–
Simmons_SAS 2024	243	0.88	–	–	–	–
Simmons_VSA 2024	243	0.85	–	–	–	–
Solomon 2020	3498	–	–	–	–	0.81
Sun_GBDT 2023	1152	–	–	0.338	0.922	–
Sun_LogReg 2023	1152	–	–	0.769	0.622	–
Sun_RF 2023	1152	–	–	0.362	0.914	–
Sun_XGBoost 2023	1152	–	–	0.339	0.907	–
Taneja 2017	444	–	–	–	–	0.81
Tzelves 2022	239	0.87	–	–	–	–
Vaid 2023	130000	–	–	0.39	0.9	–
Wang 2021	2084	–	0.668	–	–	0.654
Yang 2020	690	–	0.8764	–	–	–
Yelin 2019	315047	0.7	–	–	–	–
Yoon 2020	319	–	0.935	–	–	0.92
Zeng 2023	14177	–	–	–	–	0.85
Zhang_DL 2024	437	–	–	0.815	–	0.83
Zhang_DRF 2024	437	–	–	0.806	–	0.8
Zhang_GBM 2024	437	–	–	0.798	–	0.812
Zhang_GLM 2024	437	–	–	0.742	–	0.734
Zhang_LASSO 2024	437	–	–	0.799	–	0.799
Zhang_overall 2024	437	0.83	–	–	–	–

Ten studies were excluded at the full-text stage for the following reasons: inappropriate or irrelevant outcome measures (n = 5), absence of a true AI intervention (n = 1), or ineligible study design such as simulation studies or narrative reviews (n = 4). No studies were classified as ongoing or awaiting assessment.

### Reviewer agreement and inter-rater reliability

The screening process demonstrated high inter-reviewer agreement, confirming the robustness and reliability of study selection: title screening: 3,146 of 3,296 records agreed upon (κ = 0.911, Cohen’s kappa statistic); abstract screening: 429 of 472 records agreed upon (κ = 0.818); full-text screening: 55 of 60 records agreed upon (κ = 0.833). These kappa statistics indicate excellent agreement beyond chance, reflecting consistent application of inclusion criteria.

### Characteristics of included studies

#### Specialty distribution.

The 50 included studies encompassed a diverse range of medical specialties, reflecting the broad applicability of predictive AI-based CDSS across clinical domains. The distribution was as follows: Infectious Diseases (n = 14), Cardiology (n = 8), Emergency Medicine (n = 5), Oncology (n = 4), Gastroenterology (n = 2), Obstetrics and Gynaecology (n = 2), Orthopaedics (n = 2), Otolaryngology (n = 2), Paediatrics (n = 2), Renal (n = 2), and one study each in Dermatology, Endocrinology, Haematology, Plastic Surgery, Respiratory, Urology, and Vascular specialties.

This distribution indicates that research on predictive AI-CDSS spans multiple clinical fields, with concentration in areas characterized by high data availability, complex decision-making requirements, and urgent clinical needs.

### Geographic distribution

Studies were conducted across multiple countries, demonstrating global interest in AI-CDSS development and validation: USA (n = 16), China (n = 10), South Korea (n = 3), Israel (n = 3), Canada (n = 2), Australia (n = 2), Sweden (n = 2), Greece (n = 2), United Kingdom (n = 2), and one study each from Colombia, Germany, Hong Kong, Ireland, Italy, Japan, Thailand, and Turkey.

### *AI tool characteristics* (Table 3)

The AI models evaluated ranged from traditional machine learning algorithms (logistic regression, random forests, gradient boosting machines, support vector machines) to complex deep learning approaches (neural networks, convolutional neural networks for imaging, recurrent neural networks). Approximately one-third of studies involved imaging data (e.g., radiology or pathology images interpreted by AI), another third focused on electronic health record (EHR) tabular data or vital signs, and the remainder used other data types including genomic data, clinical text, and multimodal inputs.

**Table 3:** Summary of Studies Using AI Models and Explainability Tools.

Study Name	AI Model Type	Validation Strategy	Explainability Tool(s)	Bias Impact
Bang 2022	‘Deep learning’	Prospective validation	None	Not assessed
Bertsimas 2021	Optimal Classification Trees (OCT)	4:1 train/test split	None	Not assessed
Bhagawati 2024	Deep Neural Network	Internal validation	SHAP, LIME	Not assessed
Bolton 2024	ML predictor	retrospective hold-out	SHAP	Evaluated – the model was “not biased to individuals’ protected characteristics”
Cha 2019	CNN with radiomic features	Leave-one-out cross-validation	None	Not assessed
Connor 2007	Logistic Regression, Decision Trees	Cross-validation	None	Not assessed
Corbin 2022	Random Forest	External validation	SHAP	Not assessed
Du 2022	LightGBM	5-fold cross-validation (internal)	SHAP	Found and corrected a bias: a lower decision threshold was required for non-white patients to achieve unbiased performance
Feretzakis 2021	Random Forest	Internal validation	None	Not assessed
Gomez 2024	CNN-based ensemble	User study	SHAP	Not directly assessed
Gomez-Cabello 2024	Large Language Model (LLM, BERT-based)	External validation	None	Not assessed
Han 2020	Random Forest, Neural Network	Cross-validation	None	Not assessed
Hebert 2020	Logistic Regression, Decision Tree	Cross-validation	None	Not assessed
Hirosawa 2024	Deep Neural Network	Internal validation	None	Not assessed
Hoffer 2024	Random Forest, XGBoost	External validation	None	Not assessed
Hou 2020	XGBoost	Internal validation	None	Not assessed
Jia 2024	CNN	External validation	SHAP	Not assessed
Kanjilal 2020	Decision Tree	Internal validation	None	Not assessed
Keim-Malpass 2024	Random Forest, Gradient Boosting	Prospective validation	None	Not assessed
Lamping 2018	Random Forest, Logistic Regression	Internal validation	None	Not assessed
Lee 2021	XGBoost (not Decision Tree)	External validation (based on multi-center cohort)	None	Not assessed
Letterie 2020	Neural Network	Internal validation	None	Not assessed
Lewin-Epstein 2021	Logistic Regression, Gradient Boosting	External validation	None	Not assessed
Li 2023	XGBoost	Internal validation	SHAP	Not assessed
Liang 2022	Random Forest	Cross-validation	None	Not assessed
Liu 2023	XGBoost, Random Forest	Internal validation	None	Not assessed
McGonagle 2023	XGBoost	Prospective validation	None	Not assessed
Nau 2020	Extreme Gradient Boosting (XGBoost)	Internal validation	None	Not assessed
Oonsivilai 2019	Random Forest	External validation	None	Not assessed
Papachristou 2024	Deep CNN	Cross-validation	None	Not assessed
Pearce 2019	Logistic Regression, Gradient Boosting	External validation	None	Not assessed
Prelaj 2022	Gradient Boosting Machines (XGBoost)	Internal validation	SHAP	Not assessed
Rawson 2021	Case-based Reasoning System	Prospective validation	None	Not assessed
Rich 2022	XGBoost, Logistic Regression	Internal validation	None	Not assessed
Rojas 2024	XGBoost	External validation	SHAP	Not directly assessed
Sadik 2006	Rule-based Expert System	Retrospective validation	None	Not assessed
Shahryari Fard 2024	CNN	Cross-validation	None	Not assessed
Sick-Samuels 2020	Decision Tree	Internal validation	None	Not assessed
Simmons 2024	Logistic Regression, Decision Tree	External validation	None	Not assessed
Solomon 2020	Random Forest, SVM	Cross-validation	None	Not assessed
Sun 2023	XGBoost, Logistic Regression	Internal validation	None	Not assessed
Taneja 2017	SVM & tree-based models	Internal validation	None	Not assessed
Tzelves 2022	Random Forest	Internal 70/30 split for validation	None	Not assessed
Vaid 2023	Logistic Regression, Random Forest	Simulation study	None	Highlighted bias potential
Wang 2021	XGBoost, SVM	Internal validation	SHAP	Not assessed
Yang 2020	SVM	Cross-validation	None	Not assessed
Yelin 2019	Logistic Regression	External validation	None	Not assessed
Yoon 2020	Ensemble Model	Internal validation	None	Not assessed
Zeng 2023	CNN	External validation (SEER database)	SHAP	Not assessed
Zhang 2024	AutoML (ensembles of XGBoost, LightGBM)	Internal validation	SHAP	Not assessed

### Clinical implementation and deployment context

Of the 50 included studies, the majority (76%) evaluated AI-CDSS using retrospective clinical datasets, while 12 studies (24%) involved prospective clinical deployment or real-time implementation in healthcare settings.

Among studies reporting clinical implementation details (n = 18), the following metrics were documented: workflow integration: 11 studies described integration approaches, including EHR embedding (n = 7), standalone dashboard systems (n = 3), and mobile applications (n = 1); clinician adoption/usage rates: Reported in 6 studies, ranging from 45% to 89% of eligible cases; alert response metrics: 4 studies documented alert override rates (range: 12%–38%); time-to-decision: 3 studies reported decision time improvements (reductions of 2.3–15.7 minutes); clinical outcome measures: 8 studies assessed impacts on patient outcomes, including mortality (n = 4), length of stay (n = 3), and diagnostic accuracy in practice (n = 5)

However, the majority of studies (n = 32, 64%) focused exclusively on technical performance metrics without reporting clinical workflow integration or adoption data, representing a significant gap between ML model development and clinical utility assessment.

### Meta-analysis overview

The meta-analysis incorporated 58 outcome measurements from the 50 included studies (some studies contributed multiple outcomes) assessing the diagnostic performance of predictive AI-based clinical decision support tools. A random-effects inverse variance model using DerSimonian-Laird estimates for between-study variance (τ²) was employed.

### Diagnostic performance measures

The pooled analysis yielded the following performance metrics: Area Under the Curve (AUC): 0.652 (95% CI: 0.562–0.743), based on 58 outcome measurements; z = 14.162, p < 0.001; Specificity: 0.819 (95% CI: 0.793–0.844), based on 34 studies; Sensitivity: 0.660 (95% CI: 0.535–0.785), based on 40 studies; Accuracy: 0.765 (95% CI: 0.734–0.796), based on 39 studies. These results are visualized in forest plots (Figs 3–6).

**Fig 2:**
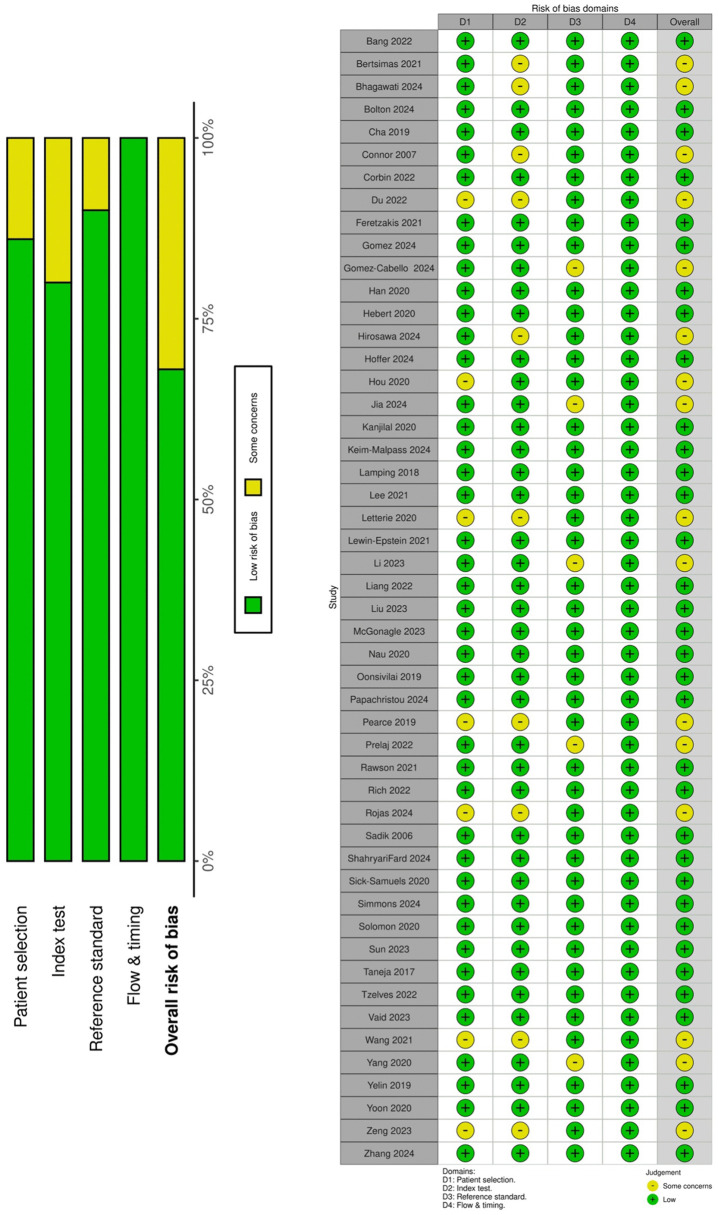
QUADAS-2 risk of bias assessment.

**Fig 3:**
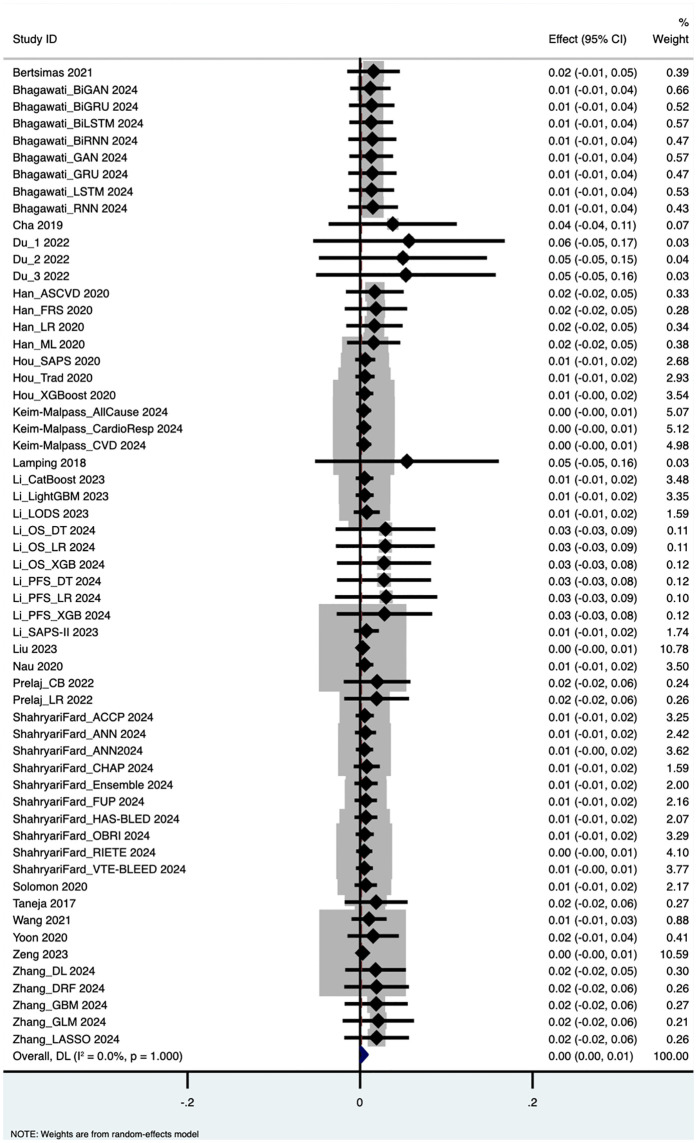
Area under the curve.

**Fig 4:**
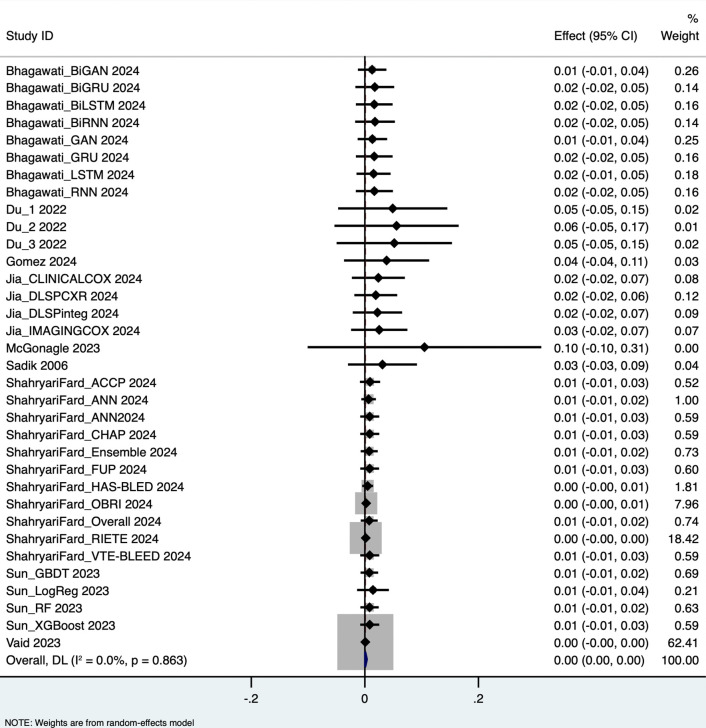
Specificity.

**Fig 5:**
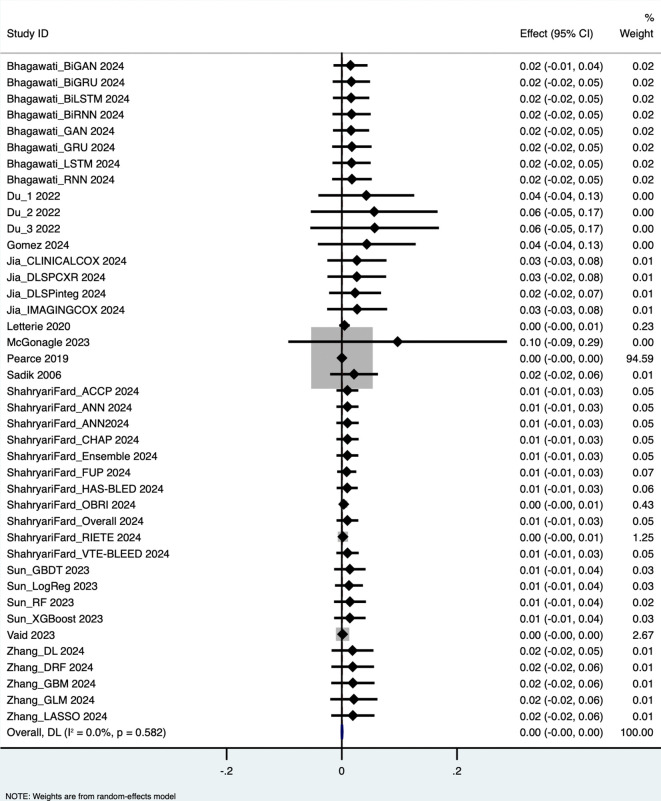
Sensitivity.

**Fig 6:**
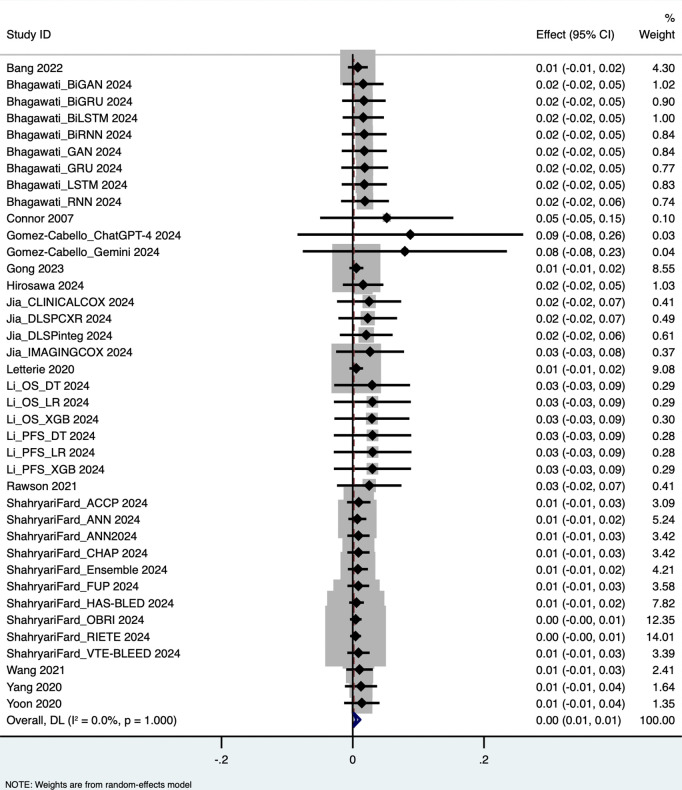
Accuracy.

### Heterogeneity and variability across studies

Substantial heterogeneity was observed across all performance metrics, confirming extreme variability unlikely to be due to chance alone: AUC Heterogeneity: Cochran’s Q = 1.2 × 10⁵; H = 46.414; I² = 100%; τ² = 0.1227; Specificity Heterogeneity: Q = 7,107.85; H = 14.676; I² = 99.5%; τ² = 0.0054; Sensitivity Heterogeneity: Q = 2.7 × 10⁵; H = 83.015; I² = 100%; τ² = 0.1624; Accuracy Heterogeneity: Q = 3,354.90; H = 9.396; I² = 98.9%; τ² = 0.0092.

### Explainability and model transparency

Among the 50 studies reviewed, 13 (26%) incorporated explainability tools to enhance model transparency. Specifically, 12 studies used SHAP (Shapley Additive Explanations), and 1 study used both SHAP and LIME (Local Interpretable Model-Agnostic Explanations). These methods were applied post hoc and did not influence the underlying predictive performance. Our analysis found no consistent differences in AUROC or accuracy between models with or without reported use of explainability tools, as these techniques are model-agnostic and do not modify algorithm outputs. Explainability tools improved model transparency and aided in identifying potential sources of bias but were insufficient for bias mitigation on their own. It should be noted that transparency is not the same as safety since SHAP and LIME alone do not mitigate bias, nor guarantee generalisability.

### Risk of Bias Assessment (QUADAS-2)

The QUADAS-2 tool (Quality Assessment of Diagnostic Accuracy Studies) [13] was employed to evaluate the methodological quality and risk of bias across included studies. Fig 2 presents a summary of QUADAS-2 risk of bias assessments for all studies. Methodological quality was heterogeneous, with most studies demonstrating at least one domain rated as high or unclear risk of bias.

Regarding patient selection, approximately 85% of studies were rated as low risk, employing consecutive patient recruitment or random sampling from existing databases to ensure representative samples. The remaining 15% exhibited unclear or high risk attributable to case-control designs or convenience sampling methodologies. For the index test domain, most studies provided comprehensive descriptions of their AI algorithms and implementation protocols. However, approximately 20% demonstrated unclear risk due to insufficient documentation of model training procedures, validation protocols, or threshold selection criteria. Concerning reference standards, the majority of studies utilized appropriate gold standards, including laboratory-confirmed diagnoses, expert consensus determinations, or validated clinical outcomes. Approximately 15% showed unclear or high risk resulting from suboptimal reference standards or inadequate blinding procedures. In the flow and timing domain, approximately 70% of studies achieved low risk ratings, with complete or near-complete patient accounting and consistent application of reference standards. The remaining 30% exhibited concerns including substantial attrition, exclusion of indeterminate results, or differential assessment timing, factors potentially introducing bias if inadequately addressed. Overall applicability was satisfactory regarding patient populations and index tests in relation to the review objectives. Given this review’s intentionally broad, multi-domain scope, most studies were deemed applicable to the research question.

The AI-specific bias evaluation revealed several critical limitations. External validation was conducted in only 32% of studies (16/50). Explainability methods, such as SHAP or LIME, were implemented in 28% of studies (14/50). Algorithmic bias was explicitly assessed in merely 4% of studies (2/50; Bolton 2024, Du 2022). Prospective validation was performed in 8% of studies (4/50).

An important distinction exists between domain-specific and global bias assessment. Re-analysis revealed that only 15 studies (30%) demonstrated low risk of bias across all four QUADAS-2 domains (patient selection, index test, reference standard, and flow/timing). While approximately 85% achieved low risk in patient selection alone, this metric does not reflect overall methodological quality. High-risk AI-specific concerns were prevalent: approximately 70% of studies (35/50) lacked external validation, 30% (15/50) demonstrated flow and timing concerns, and 20% (10/50) provided insufficient detail regarding model training or threshold determination.

A substantial gap exists between technical validation and real-world implementation evidence. Technical validation alone characterised 92% of studies (46/50), while only 8% (4/50) reported implementation outcomes. Critical metrics remained largely unreported: no studies documented adoption rates, one study reported alert override rates, no studies measured time-to-decision, and only two studies assessed patient outcomes beyond diagnostic accuracy.

### Interpretation and implications

The pooled analysis demonstrates technically promising performance but limited evidence of real‑world impact, particularly regarding specificity (0.819) and accuracy (0.765). However, sensitivity (0.660) and discriminatory ability as measured by AUC (0.652) were more moderate. The substantial heterogeneity across all metrics (I² > 98.9% for all measures) underscores considerable variability in study populations, clinical tasks, AI methodologies, and evaluation approaches. The limited reporting of clinical implementation metrics in the majority of studies highlights a critical gap between technical performance validation and real-world clinical utility assessment. While ML performance metrics provide essential information about model accuracy, they do not fully capture whether AI-CDSS tools integrate effectively into clinical workflows, achieve clinician adoption, or improve patient outcomes in practice. These findings emphasize the need for standardized evaluation methodologies, more comprehensive reporting of both technical and clinical metrics, and further validation in diverse clinical environments to enhance the generalizability and practical utility of predictive AI-CDSS tools.

## Discussion

### Principal findings

This systematic review and meta-analysis of 50 studies across 17 medical specialties revealed moderate-to-good predictive performance of AI-based clinical decision support systems, with notable variability across performance metrics and substantial methodological heterogeneity. Specificity was notably high at 81.9% (95% CI: 0.793–0.844), indicating strong potential for accurately identifying true negatives and reducing unnecessary interventions. Accuracy was robust at 76.5% (95% CI: 0.734–0.796), highlighting overall reliability in practical applications. However, sensitivity was more moderate at 66% (95% CI: 0.535–0.785), suggesting limitations in identifying true positives and raising concerns about missed diagnoses. The pooled AUC of 0.652 (95% CI: 0.562–0.743) demonstrated moderate discriminatory ability across diverse clinical contexts. Our analysis prioritised clinically meaningful discrimination metrics: primary outcomes included sensitivity, specificity, and AUROC (reported for all studies), with secondary outcomes of PPV and NPV where available. Critically, the absence of calibration assessments and prediction-decision curves in most studies represents a significant evaluation gap. While discrimination metrics reveal whether models distinguish between outcome classes, they fail to assess whether predicted probabilities align with actual outcome frequencies (essential for clinical decision-making, particularly for rare but high-consequence events where miscalibration can have catastrophic implications). We strongly advocate that future AI-CDSS evaluations adopt calibration curves and prediction-decision analyses as mandatory reporting elements, alongside explicit acknowledgment that accuracy alone may be misleading in imbalanced datasets. These tools are indispensable for evaluating whether AI systems provide actionable, reliable probability estimates that support clinical judgment, especially in scenarios where false negatives carry severe consequences.

The substantial heterogeneity observed across all performance metrics reflects the diversity of clinical tasks, patient populations, AI methodologies, and evaluation approaches encompassed in this review. This variability underscores that AI-CDSS performance is highly context-dependent and cannot be easily generalized across clinical domains without careful consideration of task-specific characteristics and implementation settings. Our meta-analysis employed the DerSimonian-Laird random-effects model, which may underestimate between-study variance under conditions of extreme heterogeneity. While alternative estimators such as restricted maximum likelihood (REML) or Paule-Mandel, potentially with Hartung-Knapp adjustments, might provide more conservative confidence intervals, we maintained the DL approach for consistency with established systematic review methodology. The substantial heterogeneity observed (I² > 90%) reflects genuine diversity in clinical domains, AI methodologies, and patient populations rather than methodological limitations, reinforcing the need for domain-specific validation of AI-CDSS.

### Gap between technical performance and clinical implementation

A critical finding is the marked gap between technical validation and real-world clinical implementation. While 76% of studies evaluated AI-CDSS using retrospective datasets, only 24% involved prospective deployment. Furthermore, 64% reported exclusively on technical metrics (sensitivity, specificity, accuracy, AUROC) without documenting workflow integration, clinician adoption, or patient outcomes. Among 18 studies (36%) reporting implementation details, approaches varied considerably, including EHR embedding, standalone dashboards, and mobile applications. However, inconsistent reporting limits assessment of clinical utility beyond technical performance. This gap highlights a fundamental challenge: demonstrating high predictive accuracy in controlled settings does not guarantee successful clinical integration or improved outcomes. Transition from development to deployment requires attention to human factors, workflow compatibility, and organizational readiness, dimensions that remain understudied.

A key methodological issue is inconsistency in performance metric reporting [14,15]. Terms like AUC and AUROC were used interchangeably, often without clear definitions [16]. Some studies differentiated AUROC from AUC-PR, offering better understanding of model performance in imbalanced datasets [17,18]. Others used “AUC” ambiguously, complicating cross-study comparisons [19]. While sensitivity and specificity were generally reported consistently, they were calculated at varying probability thresholds, making direct comparisons problematic [20,21]. Threshold choice has substantial clinical implications; optimizing for high sensitivity versus specificity represents different priorities depending on context [22,23]. Predictive values (PPV and NPV), crucial for clinical decision-making, were seldom reported despite their direct clinical relevance [24,25]. This lack of standardization undermines evidence synthesis and limits generalizability [26,27]. The field would benefit from consensus guidelines on performance metric reporting, similar to TRIPOD-AI or CONSORT-AI initiatives [28,29].

### Explainability and trust in AI-CDSS

Explainability emerged as critical for clinician trust and adoption, yet remains inadequately addressed [30,31]. Complex models, particularly deep neural networks and ensemble methods, often function as “black boxes” [32,33]. This opacity leads to clinician hesitation, especially when AI outputs conflict with clinical judgment [34,35]. Lack of explainability raises legal and ethical concerns, as clinicians must justify decisions to patients, colleagues, and in medico-legal contexts [36,37]. The question of accountability remains unresolved in most healthcare systems [38,39]. Explainability techniques such as LIME and SHAP were employed in 26% of studies [40,41]. These methods identify which features most influenced model outputs [42,43]. However, explainability tools were applied retrospectively and did not consistently improve performance or adoption [44,45]. There is an important distinction between model interpretability and prediction explainability; both are needed for full integration, yet most studies addressed only the latter [46,47]. The tension between model complexity and interpretability remains fundamental [48,49]. Simpler models offer inherent interpretability but may sacrifice accuracy compared to deep learning [50,51]. Future research must balance predictive performance with explainability, potentially through hybrid models or interpretable-by-design architectures [52,53].

### Bias, fairness, and ethical concerns in AI-CDSS

AI models inherit biases from training data, potentially perpetuating healthcare disparities [54,55]. Development must prioritize diverse, representative datasets ensuring adequate representation across race, ethnicity, sex, age, socioeconomic status, and geography [56,57]. However, diverse data alone is insufficient; developers must assess performance across demographic subgroups, implement fairness-aware algorithms, and monitor deployed models for bias [58,59]. Regulatory frameworks emphasize these requirements, though standardized approaches remain underdeveloped [60,61].

Ethical implications extend beyond technical fairness to consent, transparency, and patient autonomy [62,63]. Patients should be informed when AI contributes to their care and have mechanisms to understand or contest AI-influenced decisions [64,65]. Current practice rarely includes such transparency measures [66,67].

This review represents one of the first comprehensive multi-domain assessments of predictive AI-CDSS, addressing a critical literature gap [68,69]. Previous reviews focused on rule-based CDSS or single clinical domains [70,71]. By synthesizing evidence across 17 specialties, this review reveals patterns transcending individual contexts [72,73].

### ROADMAP framework for AI-driven clinical decision support systems in antimicrobial resistance management

We propose the ROADMAP framework to synthesise evidence-based principles for developing, implementing, and evaluating AI-driven CDSS addressing antimicrobial resistance challenges [74,75].

### Representative development principles

Development requires representative training datasets reflecting target population diversity [76–81].

### Outcomes and patient-centred evaluation

Evaluation must expand beyond technical performance to patient-centered outcomes including quality of life, satisfaction, treatment burden, and health equity impacts [82,83].

### Assessment requirements for clinical deployment

AI-CDSS applications demonstrate potential through pathogen resistance profiling [84], contact tracing [85], and predicting Gram-negative bacterial resistance [86]. Digital health tools reveal disparities between high and low-income countries [87], while intrinsic resistance mechanisms contribute significantly to mortality [88]. Infection risk modeling incorporates vital signs and laboratory results [89], with applications in sepsis prediction [90], diagnostics and drug discovery [91], battlefield medicine [92], and sociotechnical frameworks [93]. Adaptive learning systems provide implementation frameworks [94], while AI enhances biosafety protocols and outbreak management [95]. Prediction models for decompensated cirrhosis [96], nosocomial infections [97], and resistant *Enterobacterales* [98] demonstrate utility. Genomic surveillance informs vaccine development [99], though antibiotic therapy thresholds vary considerably [100]. Context-specific evaluation is essential given performance heterogeneity [101,102]. The COMBACTE-Magnet EPI-Net COACH project assembled evidence for surveillance systems [103], while European surveillance programs publish annual reports [104]. Machine learning for IV-to-oral antibiotic switches faces workflow integration and trust challenges [105]. Case-based reasoning systems demonstrate enhanced prescribing appropriateness [106], while microbiome analysis separates biological signals for AMR surveillance [107].

### Data harmonization and global surveillance

Critical gaps include limited understanding of environmental AMR levels, unclear high-risk transmission definitions, and insufficient knowledge of concentrations driving resistance [108]. These gaps are compounded by training biases, inequitable access, and standardization needs [109,110]. Global sewage analysis accommodates regional AMR diversity [111], while clinical microbiologists’ collaboration requires international consensus [112]. The TSARA trial generates actionable data on resistance and prescriptions for low-resource settings [113].

### Monitoring challenges in AMR surveillance

Ongoing monitoring addresses performance degradation, bias emergence, and evolving clinical utility [114,115]. Successful implementation encompasses workflow integration, training programs, and organizational readiness [116,117].

### Allocation of resources and economic sustainability

Rigorous health economic evaluations (cost-effectiveness analyses, budget impact assessments, value-based implementation modelling) inform resource allocation and guide sustainable integration within resource-constrained systems, fundamental to long-term viability [118,119].

### Priorities for targeted research

Critical gaps include standardized reporting frameworks, prospective validation studies in diverse settings, and implementation science frameworks elucidating determinants of AI-CDSS adoption [120,121].

### Limitations

Several limitations warrant acknowledgment. First, our focus on predictive AI-CDSS excludes knowledge graphs, natural language generation, and conversational agents [122,123]. Second, the predominance of retrospective evaluations (76%) versus prospective deployments introduces potential performance bias [124,125]. Retrospective performance often represents an upper bound for prospective deployment [126]. Third, limited reporting of implementation metrics prevented comprehensive assessment of real-world utility [127,128]. Fourth, QUADAS-2, while providing valuable quality assessment, was not designed for AI-driven diagnostic tools and may not capture AI-specific biases such as overfitting, poor generalizability, or data leakage [13,129]. The upcoming QUADAS-AI tool [130] will standardize assessment in systematic reviews [131]. Finally, our search was limited to PubMed and Cochrane Library [132]. Although these provide extensive coverage, relevant studies in computer science venues or preprint servers may have been missed [133].

## Materials and methods

This systematic review adheres to the PRISMA (Preferred Reporting Items for Systematic Reviews and Meta-Analyses) [12] guidelines to ensure transparency and methodological rigor.

### Rationale and scope

The landscape of clinical decision support has been transformed by recent advances in artificial intelligence, particularly deep learning and transformer-based architectures. While earlier systematic reviews examined traditional clinical decision support systems, the rapid evolution of AI methodologies, including convolutional neural networks, recurrent neural networks, and attention mechanisms, has created a knowledge gap requiring contemporary synthesis. Previous reviews have not comprehensively evaluated AI-based CDSS performance across multiple clinical domains using modern predictive modelling approaches, nor have they systematically assessed the standardization of performance metrics in this rapidly evolving field.

This review focuses specifically on predictive AI-based clinical decision support systems; tools that use machine learning or deep learning to generate individualized predictions or risk assessments to inform clinical decisions. This represents the largest and fastest-growing segment of AI-CDSS applications and merits focused analysis given its clinical prevalence. This operational definition intentionally excludes other AI-CDSS types such as knowledge graphs, natural language generation systems, and conversational agents, which would require different methodological approaches and are noted as a limitation of this review.

### Definition and identification of CDSS studies

To ensure consistency and reproducibility in study selection, we established explicit operational definitions. A Clinical Decision Support System (CDSS) was defined as a health information technology system designed to assist clinicians in making decisions by providing individualized, actionable recommendations or predictions based on clinical data inputs.

An AI-based predictive CDSS was defined as a digital clinical decision support tool that utilizes artificial intelligence techniques, including machine learning (ML), deep learning (DL), or natural language processing (NLP), to derive predictions or risk assessments from clinical data. This focus on predictive modeling reflects the dominant paradigm in current AI-CDSS research and clinical implementation.

Tools were excluded if they met any of the following criteria: (1) use of rule-based logic without learning algorithms (e.g., IF-THEN statements); (2) being non-digital (e.g., paper-based algorithms); (3) functioning solely as descriptive analytics tools without providing individualized outputs for clinical decision-making; or (4) representing non-predictive AI-CDSS types such as knowledge retrieval systems, natural language generation tools, or conversational agents.

### Search strategy

A systematic literature search was conducted across major biomedical databases, covering studies published up to December 6, 2024. We searched PubMed and the Cochrane Library, which were selected based on their comprehensive coverage of peer-reviewed medical literature and their established role as primary sources for clinical evidence synthesis. PubMed provides extensive indexing of biomedical journals with robust MeSH term capabilities, while Cochrane captures high-quality systematic reviews and controlled trials. For our research question focused on clinical decision support systems in healthcare settings, these databases offer comprehensive coverage of the target literature. While EMBASE provides additional European coverage, preliminary scoping indicated substantial overlap with PubMed for our inclusion criteria. IEEE Xplore, while valuable for computer science perspectives, primarily indexes technical implementations rather than clinical evaluations, which formed the core of our inclusion criteria.

The search strategy employed combinations of terms including “Clinical Decision Support System,” “CDSS,” “Artificial Intelligence,” “Machine Learning,” “Deep Learning,” “Predictive model,” and associated Medical Subject Headings (MeSH) terms. Results were supplemented with grey literature and clinical trial registries to minimize publication bias.

### Eligibility criteria

Studies were included if they met all of the following criteria:

- Tool Characteristics: The study evaluated an AI-based Clinical Decision Support System (CDSS) that used machine learning, deep learning, or related AI methods to generate individualized clinical predictions or recommendations.
- Evaluation Focus: The study assessed predictive performance using standard metrics (accuracy, sensitivity, specificity, or AUROC).
- Clinical Context: The CDSS was evaluated using real-world clinical datasets or implemented in actual healthcare settings. This included both systems deployed in clinical practice and systems rigorously validated using authentic clinical data.
- Study Design: The study used a quantitative observational or experimental design, such as retrospective cohort studies, prospective trials, or randomized controlled trials.
- Language and Publication Date: Studies published in English on or before December 6, 2024.

### Exclusion criteria

Studies were excluded if they:

- Did not use AI methodologies (e.g., relied solely on rule-based or expert systems)
- Failed to report predictive performance using standard metrics
- Were published in non-peer-reviewed formats (e.g., editorials, conference abstracts, case reports)
- Described AI tools that did not directly support clinical decision-making (e.g., image segmentation algorithms without interpretive or predictive outputs)
- Involved non-predictive AI-CDSS types (knowledge graphs, conversational agents, NLP generation systems)

### Study selection

Two reviewers (WW and AG) independently screened all retrieved records in two stages. First, they reviewed titles and abstracts to identify potentially eligible studies. Second, they conducted full-text reviews to confirm eligibility.

Discrepancies were resolved through discussion. When consensus could not be reached, a third senior reviewer (HA) made the final decision. A detailed screening log documented all decisions and rationales. Inter-rater reliability was assessed using Cohen’s kappa statistic at each stage.

### Data extraction

Data extraction was performed independently by the same two reviewers (WW and AG) using a standardized extraction template. Extracted information included: publication details: Authors, year, journal, geographic location; clinical domain and setting: Specialty, care setting (inpatient/outpatient/emergency), patient population; study characteristics: Sample size, study design, data sources; AI-CDSS characteristics: Algorithm type (e.g., random forest, neural network), input features, targeted clinical task, training dataset details; performance metrics: Sensitivity, specificity, accuracy, AUROC, PPV, NPV; clinical implementation metrics: Where reported, we extracted data on clinical workflow integration, clinician adoption rates, time-to-decision, alert override rates, and clinical outcome measures (e.g., changes in mortality, length of stay, diagnostic accuracy in practice). Any disagreements encountered during data extraction were resolved through discussion or, when necessary, through consultation with the third reviewer (HA).

### Risk of bias assessment

The QUADAS-2 tool (Quality Assessment of Diagnostic Accuracy Studies) [13] was applied to assess the methodological quality and risk of bias in the included studies. QUADAS-2 evaluates risk of bias in four domains: patient selection, index test, reference standard, and flow of patients/timing of assessments. Two reviewers independently applied QUADAS-2 to each study, with disagreements resolved by consensus.

Signalling questions were answered per QUADAS-2 guidance, and each domain was rated as “low,” “high,” or “unclear” risk of bias. We also evaluated concerns regarding applicability in each domain. It is important to note that QUADAS-2 was not originally designed to assess AI-driven diagnostic tools, which often have unique sources of bias such as overfitting to training data, poor generalizability across populations, and data leakage. As such, this quality assessment may not fully capture AI-specific bias issues. The overall QUADAS-2 ratings for each study are presented in Fig 1a and 1b.

### Outcome measures and standardization

For consistency, we standardized the definitions of key metrics across studies: Sensitivity (recall): The proportion of true positive cases correctly identified by the AI tool; Specificity: The proportion of true negative cases correctly identified; PPV (precision): The probability that a positive prediction by the AI is a true positive; NPV: The probability that a negative prediction is a true negative; Accuracy: The overall proportion of correct classifications (true positives plus true negatives over all cases); AUC (AUROC): The Area Under the Receiver Operating Characteristic curve, which plots sensitivity versus (1–specificity).

### Data synthesis and analysis

We summarized key findings of included studies qualitatively and, where appropriate, quantitatively via meta-analysis. For studies sufficiently homogeneous in terms of reported metrics, we pooled performance measures using random-effects meta-analysis models (DerSimonian-Laird method). We chose a random-effects model a priori given the anticipated heterogeneity in study populations, clinical tasks, and AI models.

Pooled estimates with 95% confidence intervals (CI) were computed for the primary metrics of interest (sensitivity, specificity, accuracy, and AUROC). Each study’s contribution was weighted by the inverse of its variance (incorporating sample size and outcome prevalence), so that larger studies (with more precise estimates) had greater influence on the pooled result.

We assessed statistical heterogeneity using Cochran’s Q and the I² statistic, with I² > 75% indicating substantial heterogeneity. The I² statistic is used to quantify the dispersion of effect sizes in a meta-analysis, representing the percentage of total variation across studies that is due to heterogeneity rather than chance; values of 25%, 50%, and 75% are commonly interpreted as low, moderate, and high heterogeneity, respectively. We also report τ² as the between-study variance. All meta-analyses were conducted using Stata Statistical Software Release 15 (StataCorp), and results are displayed in forest plots (Fig 3–6).

We evaluated publication bias qualitatively (e.g., noting if only positive studies were published in certain domains) and with funnel plots for the main outcome (AUC) when ≥10 studies were available.

Where reported in source studies, we qualitatively synthesized clinical implementation metrics including workflow integration approaches, clinician adoption patterns, alert response rates, and impacts on clinical outcomes.

No formal patient or public involvement was applicable in this evidence synthesis, as it relied on previously published studies.

## Conclusion

This review identifies that predictive AI-CDSS achieve moderate diagnostic performance across diverse specialties, with particular strength in specificity [134,135]. However, substantial performance heterogeneity, predominance of retrospective studies, and limited reporting of implementation metrics highlight a critical gap between technical validation and real-world utility assessment [136,137]. To realize AI-CDSS potential in enhancing clinical decision-making and patient care, the field must transition from focusing on technical metrics toward comprehensive evaluation encompassing workflow integration, clinician adoption, patient outcomes, and health equity impacts [138,139]. This requires collaboration across AI developers, clinicians, patients, health system administrators, regulators, and policymakers to establish standardized frameworks, address ethical concerns, and develop implementation strategies facilitating successful translation from development to deployment [140,141]. As AI methodologies evolve rapidly with deep learning, transformer architectures, and foundation models, maintaining rigorous, transparent, and clinically meaningful evaluation standards will be essential [142,143]. The QUADAS-AI tool [130] represents an important step toward standardizing quality assessment [144], and its adoption should be prioritized. By addressing identified gaps, particularly the need for prospective validation, standardized reporting, and implementation-focused research, the field can move toward evidence-based integration of AI-CDSS that demonstrably improves clinical care while maintaining safety, fairness, and patient trust [145,146].

## Supporting information

S1 File PRISMA Checklist (From: Page MJ, McKenzie JE, Bossuyt PM, Boutron I, Hoffmann TC, Mulrow CD, et al. The PRISMA 2020 statement: an updated guideline for reporting systematic reviews. BMJ 2021;372:n71. https://doi.org/10.1136/bmj.n71. This work is licensed under CC BY 4.0. To view a copy of this license, visit https://creativecommons.org/licenses/by/4.0/).
(PDF)

S1 Text Final Search Strategy. (PDF)

S1 Table Systematic Review Study Characteristics. (PDF)

S2 Table Included Studies Data. (PDF)

S3 Table Summary of Studies Using AI Models and Explainability Tools. (PDF)

S4 Table All Studies Identified in Literature Search (excel spreadsheet). (CSV)
