# Supplementary material for: Performance of predictive AI-based clinical decision support systems across clinical domains: A systematic review and meta-analysis
Source: PLOS Digit Health. 2026 Mar 24;5(3):e0001310. doi: 10.1371/journal.pdig.0001310 (PMC13012507; doi:10.1371/journal.pdig.0001310)
Supplement: S1 Text — (PDF) [file pdig.0001310.s002.pdf]

F2 Final Search Strategy

Databases Searched:

- PubMed
- Cochrane Library

Date of Final Search:

- December 6, 2024

Search Strategy Used (Both Databases):

("Artificial Intelligence"[Mesh] OR "Machine Learning"[Mesh] OR "Deep Learning"[Mesh] OR "Artificial Intelligence" OR "AI" OR "Machine Learning" OR "Deep Learning")

AND

("Clinical Decision Support Systems"[Mesh] OR "Decision Support Techniques"[Mesh] OR "Predictive Models" OR "Clinical Decision Support Systems" OR "Predictive Modelling")

AND

("Accuracy" OR "Predictive Value of Tests"[Mesh] OR "Sensitivity and Specificity"[Mesh] OR "Diagnostic Accuracy" OR "Improved Diagnosis" OR "Bias")

Filters Applied:

- Language: English
- Publication Date: up to December 6, 2024
- Study Designs: Randomised Controlled Trials (RCTs), Observational Studies, Cohort Studies
