# Supplementary material for: Performance of predictive AI-based clinical decision support systems across clinical domains: A systematic review and meta-analysis
Source: PLOS Digit Health. 2026 Mar 24;5(3):e0001310. doi: 10.1371/journal.pdig.0001310 (PMC13012507; doi:10.1371/journal.pdig.0001310)
Supplement: S1 Table — (PDF) [file pdig.0001310.s003.pdf]

S3 Table: Systematic Review Study Characteristics

	Study ID	Specialty	Country	Title	Patients
1	Bang 2022	ONC	South Korea	Deep-Learning-Based Clinical Decision Support System for Gastric Neoplasms in Real-Time Endoscopy: development and Validation Study	2524
2	Bertsimas 2021	PAEDS	Turkey	Selecting Children with Vesicoureteral Reflux Who are Most Likely to Benefit from Antibiotic Prophylaxis: application of Machine Learning to RIVUR	607
3	Bhagawati 2024	CVD	Canada	Deep learning approach for cardiovascular disease risk stratification and survival analysis on a Canadian cohort.	459
4	Bolton 2024	ID	UK	Personalising intravenous to oral antibiotic switch decision making through fair interpretable machine learning	10362
5	Cha 2019	URO	USA	Diagnostic Accuracy of CT for Prediction of Bladder Cancer Treatment Response with and without Computerized Decision Support.	123
6	Connor 2007	GASTRO	Australia	The application of machine learning techniques as an adjunct to clinical decision making in alcohol dependence treatment.	66
7	Corbin 2022	ID	USA	Personalized antibiograms for machine learning driven antibiotic selection	24148
8	Du 2022	ENDO	Ireland	An explainable machine learning-based clinical decision support system for prediction of gestational diabetes mellitus.	77
9	Feretzakis 2021	ID	Greece	Machine Learning for Antibiotic Resistance Prediction: A Prototype Using Off-the-Shelf Techniques and Entry-Level Data to Guide Empiric Antimicrobial Therapy	499
10	Gomez 2024	ENT	USA	Explainable AI decision support improves accuracy during telehealth strep throat screening.	121
11	Gomez-Cabello 2024	PLAST	USA	Large Language Models for Intraoperative Decision Support in Plastic Surgery: A Comparison between ChatGPT-4 and Gemini.	32
12	Han 2020	CVD	South Korea	Machine learning based risk prediction model for asymptomatic individuals who underwent coronary artery calcium score: Comparison with traditional risk prediction approaches.	690
13	Hebert 2020	ID	USA	Prediction of Antibiotic Susceptibility for Urinary Tract Infection in a Hospital Setting	6366
14	Hirosawa 2024	CVD	Japan	Clinical decision support system using a machine learning model to assist simultaneous cardiopulmonary auscultation: open-label randomized controlled trial	384
15	Hoffer 2024	ENT	Israel	Machine Learning for Clinical Decision Support of Acute Streptococcal Pharyngitis: A Pilot Study.	54
16	Hou 2020	ED	China	Predicting 30-days mortality for MIMIC-III patients with sepsis-3: a machine learning approach using XGboost.	4559
17	Jia 2024	RESP	China	Deep learning prediction of survival in patients with heart failure using chest radiographs.	353

18	Kanjilal 2020	ID	USA	A decision algorithm to promote outpatient antimicrobial stewardship for uncomplicated urinary tract infection	3629
19	Keim-Malpass 2024	CVD	USA	Prospective validation of clinical deterioration predictive models prior to intensive care unit transfer among patients admitted to acute care cardiology wards.	10422
20	Lamping 2018	ED	Germany	Development and validation of a diagnostic model for early differentiation of sepsis and non-infectious SIRS in critically ill children - a data-driven approach using machine-learning algorithms.	58
21	Lee 2021	ID	Hong Kong	Deep learning model for prediction of extended-spectrum beta-lactamase (ESBL) production in community-onset Enterobacteriaceae bacteraemia from a high ESBL prevalence multi-centre cohort	5626
22	Letterie 2020	OBGYN	USA	Artificial intelligence in in¬†vitro fertilization: a computer decision support system for day-to-day management of ovarian stimulation during in¬†vitro fertilization.	2603
23	Lewin-Epstein 2021	ID	Israel	Predicting Antibiotic Resistance in Hospitalized Patients by Applying Machine Learning to Electronic Medical Records	4360
24	Li 2023	CVD	China	Development and Validation of Machine Learning-Based Models to Predict In-Hospital Mortality in Life-Threatening Ventricular Arrhythmias: retrospective Cohort Study	3140
25	Liang 2022	ID	China	Early prediction of carbapenem-resistant Gram-negative bacterial carriage in intensive care units using machine learning	2920
26	Liu 2023	GASTRO	China	Construction and validation of machine learning models for sepsis prediction in patients with acute pancreatitis.	1672
27	McGonagle 2023	PAEDS	USA	Evaluation of an Antimicrobial Stewardship Decision Support for Pediatric Infections.	21
28	Nau 2020	CVD	USA	A Machine Learning Based Risk Stratification Tool to Coordinate Referrals Between Inpatient Specialty and Palliative Care for Patients with Heart Failure (RP320)	5676
29	Oonsivilai 2019	ID	Thailand	Using machine learning to guide targeted and locally-tailored empiric antibiotic prescribing in a children's hospital in Cambodia	243
30	Papachristou 2024	DERM	Sweden	Evaluation of an artificial intelligence-based decision support for the detection of cutaneous melanoma in primary care: a prospective real-life clinical trial.	228
31	Pearce 2019	ED	Australia	POLAR Diversion: Using General Practice Data to Calculate Risk of Emergency Department Presentation at the Time of Consultation.	744477
32	Prelaj 2022	ONC	Italy	Real-world data to build explainable trustworthy artificial intelligence models for prediction of immunotherapy efficacy in NSCLC patients.	480
33	Rawson 2021	ID	UK	A Real-world Evaluation of a Case-based Reasoning Algorithm to Support Antimicrobial Prescribing Decisions in Acute Care	224
34	Rich 2022	ID	USA	Development of a Prediction Model for Antibiotic-Resistant Urinary Tract Infections	9990

				Using Integrated Electronic Health Records from Multiple Clinics in North-Central Florida	
35	Rojas 2024	RENAL	Colombia	Development and validation of interpretable machine learning models to predict glomerular filtration rate in chronic kidney disease Colombian patients.	29000
36	Sadik 2006	ORTHO	Sweden	A new computer-based decision-support system for the interpretation of bone scans.	200
37	ShahryariFard 2024	HAEM	Canada	A deep-learning approach to predict bleeding risk over time in patients on extended anticoagulation therapy.	2542
38	Sick-Samuels 2020	ID	USA	A Decision Tree Using Patient Characteristics to Predict Resistance to Commonly Used Broad-Spectrum Antibiotics in Children With Gram-Negative Bloodstream Infections	689
39	Simmons 2024	ORTHO	USA	Initial clinical experience with a predictive clinical decision support tool for anatomic and reverse total shoulder arthroplasty.	243
40	Solomon 2020	CVD	USA	Forecasting a Crisis: Machine-Learning Models Predict Occurrence of Intraoperative Bradycardia Associated With Hypotension.	3498
41	Sun 2023	ONC	China	Prediction models for chronic postsurgical pain in patients with breast cancer based on machine learning approaches	1152
42	Taneja 2017	ED	USA	Combining Biomarkers with EMR Data to Identify Patients in Different Phases of Sepsis.	444
43	Tzelves 2022	ID	Greece	Using machine learning techniques to predict antimicrobial resistance in stone disease patients	239
44	Vaid 2023	ED	USA	Implications of the Use of Artificial Intelligence Predictive Models in Health Care Settings : A Simulation Study.	130000
45	Wang 2021	CVD	China	Application of machine learning to predict the occurrence of arrhythmia after acute myocardial infarction.	2084
46	Yang 2020	OBGYN	China	Predictive models of hypertensive disorders in pregnancy based on support vector machine algorithm.	690
47	Yelin 2019	ID	Israel	Personal clinical history predicts antibiotic resistance of urinary tract infections	315047
48	Yoon 2020	VASC	South Korea	MED-TMA: A clinical decision support tool for differential diagnosis of TMA with enhanced accuracy using an ensemble method.	319
49	Zeng 2023	ONC	China	Development and validation of survival prediction model for gastric adenocarcinoma patients using deep learning: a SEER-based study	14177
50	Zhang 2024	RENAL	China	Automated machine learning for early prediction of acute kidney injury in acute pancreatitis.	437
