# Supplementary material for: Performance of predictive AI-based clinical decision support systems across clinical domains: A systematic review and meta-analysis
Source: PLOS Digit Health. 2026 Mar 24;5(3):e0001310. doi: 10.1371/journal.pdig.0001310 (PMC13012507; doi:10.1371/journal.pdig.0001310)
Supplement: S2 Table — (PDF) [file pdig.0001310.s004.pdf]

S4 Table: Included Studies Data

Study ID	Patients	AUROC	Accuracy	Sensitivity	Specificity	AUC
Bang 2022	2524	-	0.815	-	-	-
Bertsimas 2021	607	-	-	-	-	0.82
Bhagawati_BiGAN 2024	459	-	0.8662	0.8812	0.9178	0.929
Bhagawati_BiGRU 2024	459	-	0.8442	0.8518	0.8317	0.908
Bhagawati_BiLSTM 2024	459	-	0.8632	0.8612	0.8529	0.916
Bhagawati_BiRNN 2024	459	-	0.8322	0.8434	0.8216	0.896
Bhagawati_GAN 2024	459	-	0.8301	0.8634	0.9113	0.916
Bhagawati_GRU 2024	459	-	0.8118	0.8317	0.8532	0.895
Bhagawati_LSTM 2024	459	-	0.8288	0.8437	0.8767	0.91
Bhagawati_RNN 2024	459	-	0.801	0.8288	0.8496	0.886
Bolton 2024	10362	0.8	-	-	-	-
Cha 2019	123	-	-	-	-	0.77
Connor 2007	66	-	0.77	-	-	-
Corbin 2022	24148	0.74	-	-	-	-
Du_1 2022	77	0.86	-	0.833	0.754	0.551
Du_2 2022	77	0.69	-	0.583	0.6	0.256
Du_3 2022	77	0.687	-	0.5	0.708	0.32
Feretzakis 2021	499	0.822	-	-	-	-
Gomez 2024	121	-	-	0.65	0.77	-
Gomez-Cabello_ChatGPT-4 2024	32	-	0.56	-	-	-
Gomez-Cabello_Gemini 2024	32	-	0.28	-	-	-
Gong 2023	5017	-	0.815	-	-	-
Han_ASCVD 2020	690	-	-	-	-	0.72
Han_FRS 2020	690	-	-	-	-	0.62
Han_LR 2020	690	-	-	-	-	0.74
Han_ML 2020	690	-	-	-	-	0.78
Hebert 2020	6366	0.69	-	-	-	-
Hirosawa 2024	384	-	0.893	-	-	-
Hoffer 2024	54	-	-	-	-	-
Hou_SAPS 2020	4559	-	-	-	-	0.797
Hou_Trad 2020	4559	-	-	-	-	0.819
Hou_XGBoost 2020	4559	-	-	-	-	0.857
Jia_CLINICALCOX 2024	353	-	0.674	0.583	0.724	-
Jia_DLSPCXR 2024	353	-	0.757	0.604	0.845	-
Jia_DLSPinteg 2024	353	-	0.818	0.75	0.776	-
Jia_IMAGINGCOX 2024	353	-	0.561	0.5	0.655	-
Kanjilal 2020	3629	0.64	-	-	-	-
Keim-Malpass_AllCause 2024	10422	-	-	-	-	0.733
Keim-Malpass_CardioResp 2024	10422	-	-	-	-	0.737
Keim-Malpass_CVD 2024	10422	-	-	-	-	0.725
Lamping 2018	58	-	-	-	-	0.78
Lee 2021	5626	0.761	-	-	-	-
Letterie 2020	2603	-	0.92	0.94	-	-
Lewin-Epstein 2021	4360	0.88	-	-	-	-
Li_CatBoost 2023	3140	-	-	-	-	0.905
Li_LightGBM 2023	3140	-	-	-	-	0.901
Li_LODS 2023	3140	-	-	-	-	0.749
Li_OS_DT 2024	273	-	0.61	-	-	0.608
Li_OS_LR 2024	273	-	0.573	-	-	0.628
Li_OS_XGB 2024	273	-	0.634	-	-	0.695
Li_PFS_DT 2024	273	-	0.549	-	-	0.696

Li_PFS_LR 2024	273	-	0.524	-	-	0.511
Li_PFS_XGB 2024	273	-	0.565	-	-	0.677
Li_SAPS-II 2023	3140	-	-	-	-	0.78
Liang 2022	2920	0.91	-	-	-	-
Liu 2023	1672	-	-	-	-	0.985
McGonagle 2023	21	-	-	0.73	0.636	
Nau 2020	5676	-	-	-	-	0.809
Oonsivilai 2019	243	0.85	-	-	-	-
Papachristou 2024	228	0.96	-	-	-	-
Pearce 2019	744477	-	-	0.96	-	-
Prelaj_CB 2022	480	-	-	-	-	0.75
Prelaj_LR 2022	480	-	-	-	-	0.77
Rawson 2021	224	-	0.83	-	-	-
Rich 2022	9990	0.66	-	-	-	-
Rojas 2024	29000	-	-	-	-	-
Sadik 2006	200	-	-	0.9	0.74	-
ShahryariFard_ACCP 2024	2542	0.667	0.696	0.606	0.7	0.081
ShahryariFard_ANN 2024	2542	0.771	0.854	0.424	0.874	0.113
ShahryariFard_ANN2024	2542	0.612	0.743	0.515	0.753	0.072
ShahryariFard_CHAP 2024	2542	0.681	0.743	0.545	0.752	0.188
ShahryariFard_Ensemble 2024	2542	0.824	0.808	0.606	0.817	0.141
ShahryariFard_FUP 2024	2542	0.807	0.76	0.758	0.76	0.129
ShahryariFard_HAS-BLED 2024	2542	0.642	0.908	0.333	0.935	0.135
ShahryariFard_OBRI 2024	2542	0.663	0.944	0.03	0.986	0.08
ShahryariFard_Overall 2024	2542	-	-	0.61	0.82	-
ShahryariFard_RIETE 2024	2542	0.615	0.951	0	0.994	0.063
ShahryariFard_VTE-BLEED 2024	2542	0.651	0.74	0.485	0.752	0.069
Sick-Samuels 2020	689	0.7	-	-	-	-
Simmons_Abduction 2024	243	0.72	-	-	-	-
Simmons_ASES 2024	243	0.87	-	-	-	-
Simmons_Constant 2024	243	0.89	-	-	-	-
Simmons_Elevation 2024	243	0.74	-	-	-	-
Simmons_Global 2024	243	0.83	-	-	-	-
Simmons_IR 2024	243	0.8	-	-	-	-
Simmons_Rotation 2024	243	0.73	-	-	-	-
Simmons_SAS 2024	243	0.88	-	-	-	-
Simmons_VSA 2024	243	0.85	-	-	-	-
Solomon 2020	3498	-	-	-	-	0.81
Sun_GBDT 2023	1152	-	-	0.338	0.922	-
Sun_LogReg 2023	1152	-	-	0.769	0.622	-
Sun_RF 2023	1152	-	-	0.362	0.914	-
Sun_XGBoost 2023	1152	-	-	0.339	0.907	-
Taneja 2017	444	-	-	-	-	0.81
Tzelves 2022	239	0.87	-	-	-	-
Vaid 2023	130000	-	-	0.39	0.9	-
Wang 2021	2084	-	0.668	-	-	0.654
Yang 2020	690	-	0.8764	-	-	-
Yelin 2019	315047	0.7	-	-	-	-
Yoon 2020	319	-	0.935	-	-	0.92
Zeng 2023	14177	-	-	-	-	0.85
Zhang_DL 2024	437	-	-	0.815	-	0.83
Zhang_DRF 2024	437	-	-	0.806	-	0.8
Zhang_GBM 2024	437	-	-	0.798	-	0.812

Zhang_GLM 2024	437	-	-	0.742	-	0.734
Zhang_LASSO 2024	437	-	-	0.799	-	0.799
Zhang_overall 2024	437	0.83	-	-	-	-
