# Supplementary material for: Performance of predictive AI-based clinical decision support systems across clinical domains: A systematic review and meta-analysis
Source: PLOS Digit Health. 2026 Mar 24;5(3):e0001310. doi: 10.1371/journal.pdig.0001310 (PMC13012507; doi:10.1371/journal.pdig.0001310)
Supplement: S3 Table — (PDF) [file pdig.0001310.s005.pdf]

S5 Table: Summary of Studies Using AI Models and Explainability Tools

Study Name	AI Model Type	Validation Strategy	Explainability Tool(s)	Bias Impact
Bang 2022	'Deep learning'	Prospective validation	None	Not assessed
Bertsimas 2021	Optimal Classification Trees (OCT)	4:1 train/test split	None	Not assessed
Bhagawati 2024	Deep Neural Network	Internal validation	SHAP, LIME	Not assessed
Bolton 2024	ML predictor	retrospective hold-out	SHAP	Evaluated – the model was "not biased to individuals' protected characteristics"
Cha 2019	CNN with radiomic features	Leave-one-out cross-validation	None	Not assessed
Connor 2007	Logistic Regression, Decision Trees	Cross-validation	None	Not assessed
Corbin 2022	Random Forest	External validation	SHAP	Not assessed
Du 2022	LightGBM	5-fold cross-validation (internal)	SHAP	Found and corrected a bias: a lower decision threshold was required for non-white patients to achieve unbiased performance
Feretzakis 2021	Random Forest	Internal validation	None	Not assessed
Gomez 2024	CNN-based ensemble	User study	SHAP	Not directly assessed
Gomez-Cabello 2024	Large Language Model (LLM, BERT-based)	External validation	None	Not assessed
Han 2020	Random Forest, Neural Network	Cross-validation	None	Not assessed
Hebert 2020	Logistic Regression, Decision Tree	Cross-validation	None	Not assessed
Hirosawa 2024	Deep Neural Network	Internal validation	None	Not assessed
Hoffer 2024	Random Forest, XGBoost	External validation	None	Not assessed
Hou 2020	XGBoost	Internal validation	None	Not assessed
Jia 2024	CNN	External validation	SHAP	Not assessed
Kanjilal 2020	Decision Tree	Internal validation	None	Not assessed
Keim-Malpass 2024	Random Forest, Gradient Boosting	Prospective validation	None	Not assessed
Lamping 2018	Random Forest, Logistic Regression	Internal validation	None	Not assessed
Lee 2021	XGBoost (not Decision Tree)	External validation (based on multi-center cohort)	None	Not assessed
Letterie 2020	Neural Network	Internal validation	None	Not assessed

Lewin-Epstein 2021	Logistic Regression, Gradient Boosting	External validation	None	Not assessed
Li 2023	XGBoost	Internal validation	SHAP	Not assessed
Liang 2022	Random Forest	Cross-validation	None	Not assessed
Liu 2023	XGBoost, Random Forest	Internal validation	None	Not assessed
McGonagle 2023	XGBoost	Prospective validation	None	Not assessed
Nau 2020	Extreme Gradient Boosting (XGBoost)	Internal validation	None	Not assessed
Oonsivilai 2019	Random Forest	External validation	None	Not assessed
Papachristou 2024	Deep CNN	Cross-validation	None	Not assessed
Pearce 2019	Logistic Regression, Gradient Boosting	External validation	None	Not assessed
Prelaj 2022	Gradient Boosting Machines (XGBoost)	Internal validation	SHAP	Not assessed
Rawson 2021	Case-based Reasoning System	Prospective validation	None	Not assessed
Rich 2022	XGBoost, Logistic Regression	Internal validation	None	Not assessed
Rojas 2024	XGBoost	External validation	SHAP	Not directly assessed
Sadik 2006	Rule-based Expert System	Retrospective validation	None	Not assessed
Shahryari Fard 2024	CNN	Cross-validation	None	Not assessed
Sick-Samuels 2020	Decision Tree	Internal validation	None	Not assessed
Simmons 2024	Logistic Regression, Decision Tree	External validation	None	Not assessed
Solomon 2020	Random Forest, SVM	Cross-validation	None	Not assessed
Sun 2023	XGBoost, Logistic Regression	Internal validation	None	Not assessed
Taneja 2017	SVM & tree-based models	Internal validation	None	Not assessed
Tzelves 2022	Random Forest	Internal 70/30 split for validation	None	Not assessed
Vaid 2023	Logistic Regression, Random Forest	Simulation study	None	Highlighted bias potential
Wang 2021	XGBoost, SVM	Internal validation	SHAP	Not assessed
Yang 2020	SVM	Cross-validation	None	Not assessed
Yelin 2019	Logistic Regression	External validation	None	Not assessed
Yoon 2020	Ensemble Model	Internal validation	None	Not assessed
Zeng 2023	CNN	External validation (SEER database)	SHAP	Not assessed

Zhang 2024	AutoML (ensembles of XGBoost, LightGBM)	Internal validation	SHAP	Not assessed
